# Oncolytic immunovirotherapy for high-grade gliomas: A novel and an evolving therapeutic option

**DOI:** 10.3389/fimmu.2023.1118246

**Published:** 2023-03-15

**Authors:** Sweety Asija, Abhishek Chatterjee, Jayant S. Goda, Sandhya Yadav, Godhanjali Chekuri, Rahul Purwar

**Affiliations:** ^1^ Department of Biosciences, Indian Institute of Technology, Mumbai, India; ^2^ Department of Radiation Oncology, Tata Memorial Centre (TMH & ACTREC) & Homi Bhabha National Institute, Mumbai, India

**Keywords:** immunovirotherapy, oncolytic virus, high-grade, gliomas, anti-tumor immunity

## Abstract

Glioblastoma is one of the most difficult tumor types to manage, having high morbidity and mortality with available therapies (surgery, radiotherapy and chemotherapy). Immunotherapeutic agents like Oncolytic Viruses (OVs), Immune Checkpoint Inhibitors (ICIs), Chimeric Antigen Receptor (CAR) T cells and Natural Killer (NK) cell therapies are now being extensively used as experimental therapies in the management of glioblastoma. Oncolytic virotherapy is an emerging form of anti-cancer therapy, employing nature’s own agents to target and destroy glioma cells. Several oncolytic viruses have demonstrated the ability to infect and lyse glioma cells by inducing apoptosis or triggering an anti-tumor immune response. In this mini-review, we discuss the role of OV therapy (OVT) in malignant gliomas with a special focus on ongoing and completed clinical trials and the ensuing challenges and perspectives thereof in subsequent sections.

## Graphical Abstract

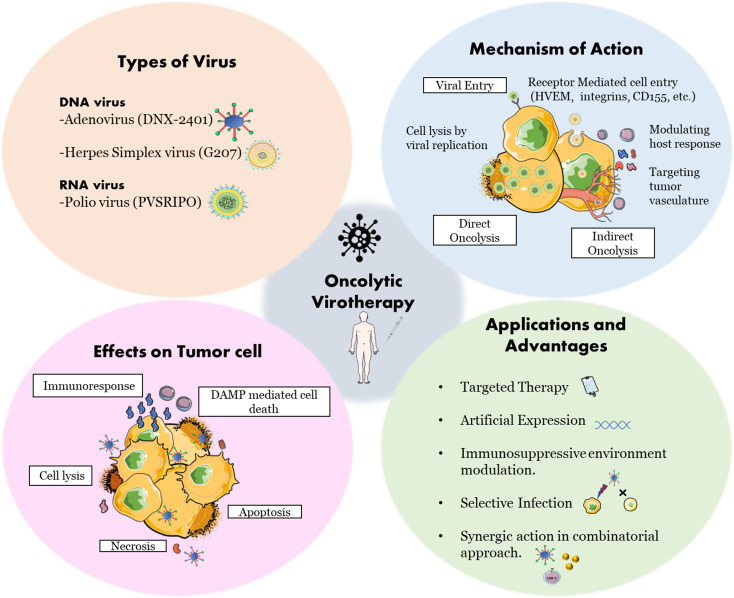

## Introduction

Glioblastoma (GBM) is the most common adult primary brain tumor characterized by aggressive behavior, ubiquitous progression, and fatal outcomes in the majority of patients despite aggressive treatment strategies incorporating surgery, radiotherapy, and chemotherapy (1).

In recent years, sophisticated, personalized, and targeted immunotherapeutic approaches have emerged as novel therapeutics in the armamentarium against glioblastoma. This has been due to the gradual erosion of the canonical assumption of the brain as an immune-privileged organ (2). Widely used immunotherapy treatments include monoclonal antibodies, checkpoint blockade inhibitors, cancer vaccines, adoptive cell transfer, dendritic cell vaccines, and CAR-T cells. However, gliomas typically display immunologically cold signatures with limited immune cells available that are needed for an immune attack on the tumor (3). Consequently, the initial encouraging data has not translated into survival benefits in randomized trials (4) and significantly tempered initial optimism. These setbacks spur the development of newer immunotherapeutic approaches striking the right balance between efficacy and toxicity. Oncolytic Virotherapy (OVT) is one such exciting new approach on the horizon in personalized glioma therapy.

## Oncolytic viruses

Oncolytic viruses are lab-grown attenuated viruses that exhibit the ability to target and eradicate tumor cells while mimicking natural growth processes. An artificially attenuated replication-competent virus was made feasible by implementing the confluence of modern strategies including molecular, cellular, and cloning engineering with the greater understanding of the genetic makeup of viruses (5).

**Figure 1:**
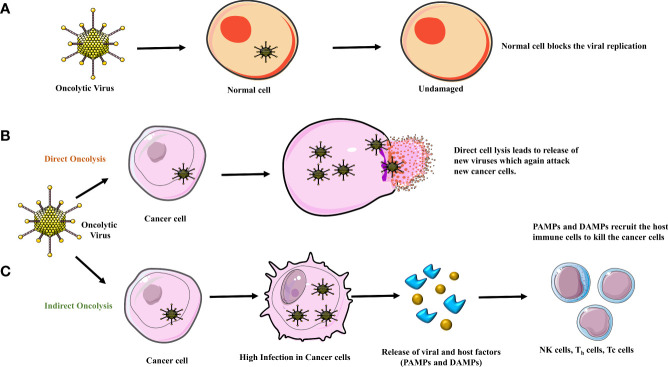
**(A)** Normal cells contain an anti-viral response system blocking the replication of the oncolytic virus, leaving them unharmed. Oncolysis occurs in two ways, **(B)** when the viral load in the tumor cell increases there is direct cell lysis. **(C)** In an indirect way, due to high infection in the tumor cells, DAMPs (damage-associated molecular pattern) and PAMPs (pathogen-associated molecular pattern) are released due to which the major histocompatibility complex (MHC) and Antigen Presenting Cell (APC) recruit the host immune cells to attack the cancer cell leading to cell death/apoptosis.

With continuous enhancements in efficacy and safety profiles, viruses may be used to treat cancer. These specially crafted viruses are designed to be non-pathogenic and can enter the host environment through receptors and specifically target cancer cells. After the entry, anticancer immune responses and tumor lysis is activated (**Figure 1**). These viruses can send out warning signals prompting the body’s immune system to launch antitumor responses. The *ex-vivo* nature of immunomodulation, coupled with the availability of suitable vectors with neurotropism make gliomas an attractive option for OVT. In addition, OVT has additional advantages over current glioma therapies including the capacity for direct neural cell invasion, apoptosis-independent cell lysis, activation, and recruitment of immune cells to the brain. The subsequent sections will briefly discuss several aspects of OVT and current and future perspectives in this regard.

## Oncolytic viruses for targeted anti-glioma therapy

Replication-competent viruses are currently being employed in anti-glioma therapy. Replication-competent viruses exert their therapeutic effect through direct action by lysis of tumor cells or by indirect action *via* modulation of glioma-related apoptotic pathways (6). Moreover, replication-competent viruses are genetically engineered to conditionally replicate and amplify only in the tumor cell without productive infection of normal cells **(** **Figure 1A** **)**. Additionally, the replication-competent OVs have high transduction efficiency (7).

## Salient characteristics of oncolytic virus render them an attractive candidate for anti-glioma immunotherapy

Oncolytic viruses (OVs) have certain inherent features rendering them attractive options as *de-novo* immunotherapy in GBM. These characteristics include:

1. Non-pathogenic nature with a favorable safety profile as a genetic modification vector.
2. Wide-ranging tumor cell infectiousness.
3. Good patient tolerance with high capability for intra-tumoral replication.
4. Wide applicability across multiple tumor types.
5. Synergistic and cumulative effects with conventional and other immunotherapeutic approaches.
5. Ease of modification for therapeutic improvement.
6. Favorable modification of Tumor Microenvironment (TME).
7. Simultaneous targeting of multiple tumor foci as both a local and systemic therapy.

## Synthesis and augmentation of OVs

Naturally occurring viruses have DNA or RNA as genetic material with single or double strands. The size of the genomic material ranges from 2 to 300 kbps having positive or negative sense strands. This enables it to hold or integrate the transgenes from other species like microorganisms, humans or murine, etc. The transgene largely varies in size from 100 base pairs to several kilobase pairs to confer additional appealing properties to improve their applicability in the field of oncology.

Majorly, oncolytic viruses are genetically engineered, modified, and reprogrammed to improve the tumor-specific tropism and enhance immune evasion and anti-tumor efficacy with an aiming target the infected and uninfected tumor cells without causing any damage to the normal bystander cell population (8).

## Anti-cancer mechanism of OVs

**Figure 2:**
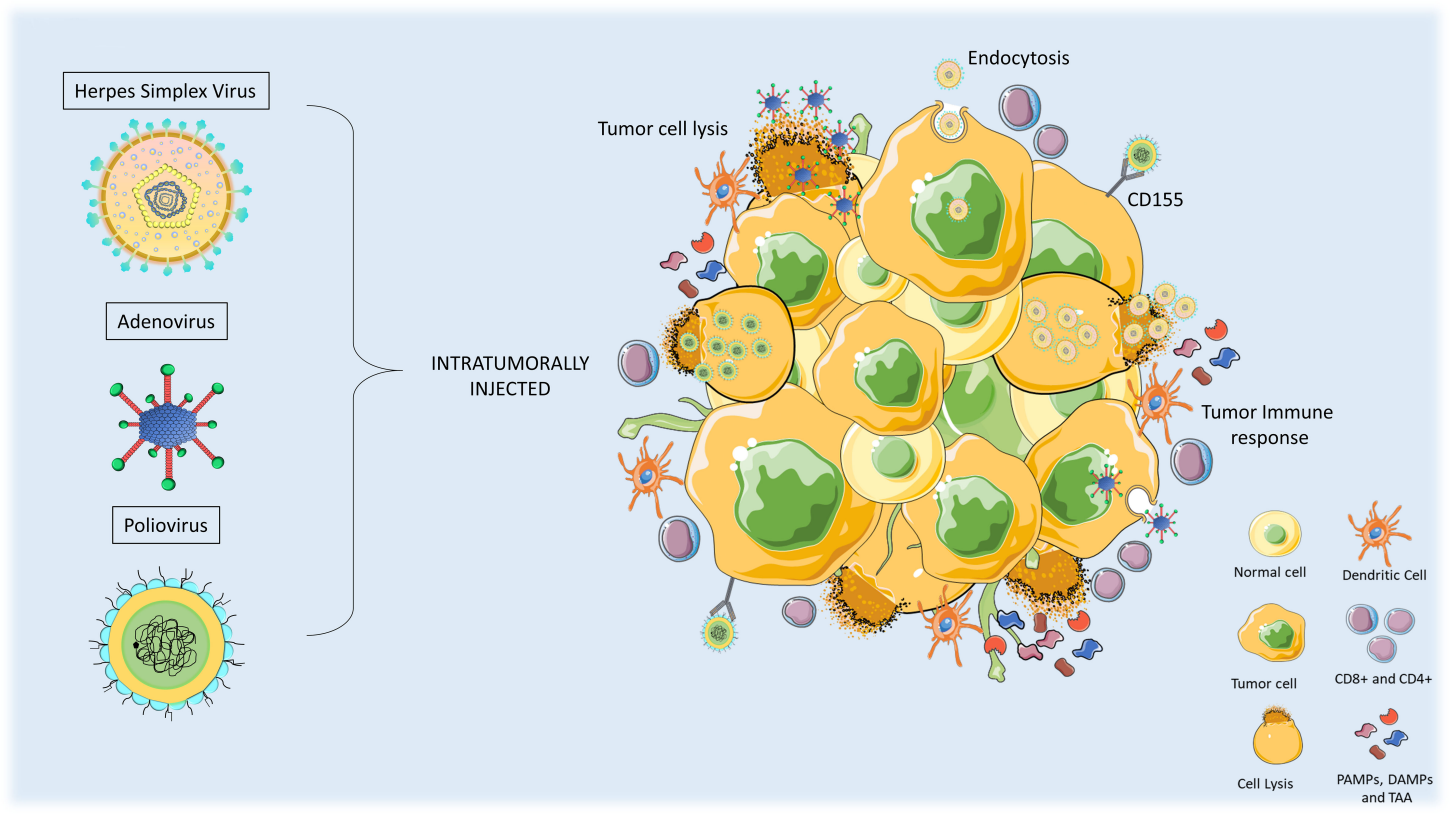
Anti-tumor mechanism of action of oncolytic viruses in high-grade gliomas (HGG). Oncolytic Viral therapies include genetic modifications in the viruses. When administered intratumorally, the virus can infect normal and tumor cells but only replicates and lyse the tumor cells. Upon oncolysis, viral progenies are released that infect other tumor cells and release pro-immunogenic factors such as PAMPs, DAMPs, and tumor-associated antigens (TAA). Innate immune response triggers activation of antigen-presenting cells (APCs) and T-cells which leads to the induction of immunogenic cell death of tumor cells.

The anticancer mechanism of the OV includes both direct oncolysis & indirect oncolysis (**Figures 1B, C**). By direct oncolysis, Oncolytic viruses selectively replicate in cancer cells and cause the inflammation and even death of cancer cells, further leading to host immune responses because of cancer-associated antigen exposure (9) (10). The indirect anti-neoplastic mechanism can be induced by the bystander effects leading to the destruction of blood vessels or by immune modulation within the tumor **(** **Figure 2** **)** (11).

## Direct action by glioma cell lysis

A suitable environment or particular conditions are required for the virus replication in neoplastic cells. Once the viruses integrate within the glioma cell, they starve the tumor cell of their growth nutrients by capturing the tumor cell protein factory. Thereafter, the normal physiological processes of the tumor cell are destroyed. Infected neoplastic cells are characterized by dysfunctional type I IFN signaling elements and low levels of protein kinases R (PKR). Under these circumstances, the virus replicates most efficiently in tumor cells. During tumor cell lysis, various molecules including soluble tumor antigens and danger-associated molecular factors are released. These factors further enhance tumor-specific immunity by priming immune cells (12).

## Indirect action: Modulating OVs-mediated host immune responses

Oncolytic viruses have the potential to reshape immunologically silent or cold tumors into hot tumors thus enhancing the sensitivity of treatment strategies (13). This can be achieved by functionally activating the lytic cycle of the oncolytic virus which involves, the disintegration of the extracellular matrix, disruption of the tumor microenvironment, and release of tumor-associated antigens (TAAs), followed by the activation of innate immune and adaptive immune responses (**Figure 2**). The activation of pathogen-associated molecular pattern sensors and toll-like receptors evoke innate immune cells including natural killer (NK) cells, granulocytes, neutrophils, and antigen-presenting cells (APCs), as a primary response. The adaptive immune system also gets triggered by dendritic cells primed with TAAs. Furthermore, infiltration of T cells in the tumor is also facilitated by the release the tumor-specific antigens (14, 15). The necrosis induced by OVs can also cause the release of damage-associated molecular patterns (DAMPs), which stimulate dendritic cells and acquired immune responses (16).

Oncolytic viruses manipulate the immunosuppressive tumor microenvironment, cause the destruction of tumor cells, help in tumor-associated antigen presentation to dendritic cells, and trafficking and survival of effector T cells at the tumor site, thereby triggering the *de-novo* anti-tumor response of T cells and re-stimulate the innate and adaptive immune system (17). The OV uses various strategies to destroy the immune-suppressive environment through arming with immune-modulating genes including genes encoding inhibitors of immune checkpoints, tumor antigens, and targets for chimeric antigen receptor T cells, to further improve overall immune responses, especially for immunologically “cold” tumors. OVs can be engineered to express modulatory molecules that target the structure of the tumor microenvironment to destroy tumor cells and impair the support for the growth of the tumor.

## Augmentation by transgene expression

1. Expression of immunomodulatory transgenes such as IL-2, for enhancing antitumor efficacy and survival (18). Additionally, IL-12 also possesses anti-cancer activity and inhibits tumor angiogenesis. Oncolytic virus encoding cytokines are engineered for their local production, however, these virus expressing cytokines are not effective as monotherapy. Furthermore, in mouse models, a dual combination of OHSV G47Δ expressing murine IL-12 (G47Δ-mIL12) along with the antibodies to immune checkpoints (CTLA-4, PD-1, PD-L1), showed survival benefits in mice. However, the triple combination of G47Δ-mIL12 anti-CTLA-4, and anti-PD-1 cured most of the mice in two glioma models. Therefore, a combinatorial approach, using OVs encoding cytokines and immunotherapy including immune checkpoint inhibitors are required to improve the anti-tumor efficacy (19–21).
2. Expression of cytolytic, and immunomodulatory transgenes-IL-15 introduction is performed to mediate tumor cell lysis which further decreases tumor size and induces innate and adaptive effector immune responses (22).
3. Expression of immune stimulants OX40 and granulocyte-macrophage colony-stimulating factor (GM-CSF) are used to accentuate the immune response against the tumor (23–25).

## Indirect action: Targeting the tumor vasculature in glioblastomas

OVs can also destroy tumor blood vessels, reducing or even disrupting tumor blood supply, leading to tumor hypoxia and lack of nutrients (26, 27). Direct interaction of OV with tumor blood vessels mainly neo vasculature results in massive cell death. The resultant killing within the tumor is characterized by irreparable tumor vasculature damage caused by the initiation of fibrin accumulation and microthrombi within blood vessels triggered by neutrophils. The extensive cell death caused by clot formation is limited to the tumor beds. A study demonstrated the role of intravascular clot formation in initiating robust antitumor efficacy by the apoptosis of tumor cells and with a decreased cell proliferation rate (28).

OVs incite chemokines which are induced by interferon which in turn activate endothelial cells and permit immune cells to enter the blood-brain barrier (29). The synergism of multiple anti-tumor pathways suggests that OVs can effectively engage in anti-glioma activity and form a component of intensive combinatorial anti-glioma strategies.

## Oncolytic viruses mediated clinical trials for the treatment of high-grade gliomas

Oncolytic viruses have been employed to treat solid tumors, mainly glioblastoma with initial encouraging results. Major classes of viruses employed for GBM treatment include adenovirus, herpes simplex virus (HSV), and poliovirus. Numerous completed and ongoing oncolytic virus therapy trials are mentioned for adenovirus, HSV, and Poliovirus (**Table 1**). OV is being implemented both as a monotherapy and also used as a combinatorial approach with other therapies, to further enhance the efficacy and antitumor activity. Here, we briefly discuss a few of the interesting features, clinical status, and outcomes of some OVs for glioblastomas **(** **Table 2** **).**

**Table 1:** Adenovirus, HSV & Poliovirus based oncolytic virus therapy for the treatment of brain tumors.

Clinical Trial no. (clinicaltrials.gov.in)	Virus type	Phase	Status	Aim	Ref.
NCT01582516 (2022)	Δ-24-RGD	I/II	Completed	Determine the safety and tolerability of Δ-24-RGD administered by Convection Enhanced Delivery in patients with recurrent GBM.	(30)
NCT03896568 (2022)	MSC-Ad5-DNX-2401	I	Recruiting	Use of perfusion guidance to enhance the precision of ESIA infusions of mesenchymal stem cells loaded with Δ-24 (MSC-D24).	(31)
NCT05084430 (2022)	M032	I/II	Active	Determine the safety and tolerability of Pembrolizumab when given in conjunction with M032, an oHSV that expresses IL-12.	(32)
NCT03072134 (2021)	NSC-CRAd-Survivin-pk7	I	Completed	Safety and efficacy of delivering a novel oncolytic adenovirus *via* a neural stem cell line in combination with radiation and chemotherapy.	(33)
NCT04758533 (2021)	AloCELYVIR	I/II	Recruiting	Assess the safety and efficacy of AloCELYVIR, which consist of bone marrow-derived allogeneic mesenchymal stem cells infected with an Adenovirus, ICOVIR-5.	(34)
NCT03152318 (2020)	rQNestin	I	Completed	Determine the maximum tolerated dose of rQNestin34.5v.2 with or without previous immunomodulation with cyclophosphamide.	(35)
NCT03657576 (2019)	C134	I	Active	Safety and efficacy of C134.	(36)
NCT03714334 (2019)	DNX-2440	I	Recruiting	Safety and efficacy of stereotactic injection of DNX-2440.	(37)
NCT02798406 (2018)	DNX-2401	II	Completed	Efficacy of single injection of DNX-2401, when delivered intratumorally followed by the administration of intravenous Pembrolizumab.	(38)
NCT00805376 (2018)	DNX-2401	I	Completed	Define the Maximum Tolerated Dose of DNX-2401 injection into brain tumors.	(39)
NCT04482933 (2017)	G207	II	Not yet recruiting	Efficacy and safety of intratumoral inoculation of G207 combined with a single 5 Gy dose of radiation.	(40)
NCT02197169 (2017)	DNX-2401	I	Completed	Efficacy of single injection of DNX-2401, when delivered intratumorally with or without subsequent interferon-gamma (IFN-γ)	(41)
NCT02986178 (2017)	PVSRIPO	II	Active	Safety and efficacy of PVSRIPO	(42)
NCT02444546 (2015)	Pelareorep	I	Active, not recruiting	To study the side effects and the best dose of wild-type reovirus (viral therapy) when given with sargramostim in high grade brain tumors	(43)
NCT00157703 (2014)	G207	I	Completed	Evaluate the safety and tolerability of intratumoral administration of G207 following radiation therapy	(44)

**Table 2:** Major clinical trials reporting the efficacy of oncolytic virus therapy for the treatment of brain tumors.

Clinical Trial no. (clinicaltrials.gov.in)	Virus type	Phase	Status	Aim	Ref.
NCT03178032 (2022)	DNX-2401	I	Active	To assess the safety and adverse-event profile of DNX-2401.	(45)
NCT02457845 (2021)	G207	I	Completed	Determine the safety of combining G207 alone or with a single low dose of radiation.	(46)
NCT01491893 (2018)	PVSRIPO	I	Completed	Determine the MTD and the Recommended Phase 2 Dose of PVSRIPO	(47)

## Initial encouraging results of oncolytic virotherapy

### DNX-2401

The US Food and Drug Administration has expedited the development of an adenovirus known as DNX-2401 (Δ-24-RGD, or tasadenoturev) for patients with malignant glioma due to the paucity of viable treatment options in relapsed or progressive disease. The virus has a deletion of 24 base pairs in a crucial gene E1A and an insertion of Arg–Gly–Asp (RGD) motif in a viral capsid protein, thereby enhancing the specificity of tumor cell targeting and with improved affinity for αV integrin. DNX-2401 demonstrated a virus-mediated mechanism of tumor cell necrosis (48). A phase I, dose-escalation clinical trial was the first to demonstrate the direct oncolysis by adenovirus in brain tumors. Post-treatment, 20% of patients survived more than 3 years and three of them had a dramatic response with >95% reduction in tumor size with long-term survival after treatment with DNX2401 (49). Encouraging early results have been obtained in combination with ICIs as well (50). Encouraging results have also been obtained in combination with Radiotherapy (RT) in pediatric Diffuse Intrinsic Pontine Glioma (DIPG) with a median survival of 17.8 months albeit with a notable toxicity burden that will require mitigation. The most common adverse events observed included neurologic deterioration, headache, and vomiting in 9 patients each, fatigue in 8 patients and fever in 6 patients. Majority of the events observed were of Grade-1 severity (14/19 events), 4/19 events were of grade2 severity while grade-3 adverse events was observed in 1 patient only. No grade-4 and 5 adverse events were observed in the study (51).. Multiple ongoing clinical trials are exploring its effectiveness in combination therapies and there is much optimism shared by researchers in this regard.

### PVSRIPO

Researchers at Duke University investigated the therapeutic value of PVSRIPO, a live chimeric attenuated poliovirus type 1 (Sabin) vaccine. PVSRIPO was delivered to 61 patients of recurrent supratentorial Grade IV malignant glioma and a safe dose of 5.0 x 10^7^ TCID50 was determined from the phase II trial. Grade -4 intracranial hemorrhage has been observed as a dose limiting adverse event at a dose level (10^10^ TCID50). To attain the optimum dose in phase II, the dose was de-escalated to reduce the loco-regional inflammation of the tumor. A grade 3 or higher PVSRIPO-related adverse event was experienced by 19% of the patients during the dose-expansion period. At a follow-up of 24 months, patients who received PVSRIPO achieved an overall survival (OS) of 21% (95% CI-11-33), which persisted up to 36 months. When PVSRIPO was infused intratumorally, there had been no reports of viral shedding or neuropathogenic changes (52).

### G207

G207, a genetically engineered Herpes Simplex Virus (HSV) type 1 in combination with low doses of RT showed encouraging responses, increased immune cell infiltration, and favorable toxicity in recurrent or progressive Pediatric High-Grade Glioma (PHGG). A phase I trial of G207 recruited twelve patients in the pediatric and adolescent age group (7 to 18 years) with a biopsy proven recurrent and progressive supratentorial brain tumors. This phase -I study had four dose cohorts. G207 (10^7^ or 10^8^ plaque-forming units) was administered intracranially through controlled infusion for 6 hours duration. Within 24 hours of G207 administration, cohorts 3 and 4 received radiotherapy (5 Gy) to the gross tumor volume. Results of the study demonstrated the absence of any dose-limiting toxicities or significant adverse events related to G207. Twenty unfavorable grade-1 incidents attributed to G207 were documented. No signs of viral shedding were observed. Eleven patients displayed radiographic, neuropathological, and clinical response. The median OS was 12.2 months (95% CI-8-16.4 months) and 4 out of 11 patients were alive for more than 18 months post-G207 therapy (53).

### DELYTACT

DELYTACT (G47Δ; teserpaturev), a triple mutant and a third-generation oncolytic virus, with the deletions of *α47* gene and overlapping *US11* promoter from parental G207, a second-generation oncolytic HSV-1 with deletions in both copies of the *γ34.5* gene and an inactivation of the *ICP6* gene (54). It became the first oncolytic virus in the world to be approved for patients with residual or recurrent glioblastoma. Preclinical evidence showed that G47Δ is effective *via* two different mechanisms: a direct oncolytic impact, an immediate effect caused by virus multiplication, and a delayed effect showing antitumor immunity. Encouraging preclinical results led investigators to do a first-in-human study of G47Δ multiple intratumoral infusions (twice within two weeks), in patients diagnosed with recurrent glioblastoma. The safety profile was analyzed and G47Δ OV was deemed to be safe. Thereafter, in a separate phase-2 study, this oncolytic HSV-1 was administered to 19 adult patients with residual or recurrent supratentorial glioblastoma. Delytact was administered intratumorally for up to six cycles, following irradiation and temozolomide treatment. The results of the phase-2 trial showed excellent response with 1-yr overall survival in 84% of patients and a median overall survival of 20.2 months post OVT initiation (16.8–23.6) (55). The treatment was also associated with a favorable safety profile (56).

## Other promising agents

### ParvOryx

ParvOryx can spread in the tumor by crossing the blood-brain barrier. Furthermore, it activates the antibody formation in a dose dependent manner resulting in T cell responses. Oncolytic H-1 parvovirus (ParvOryx) was studied in 18 patients with recurrent GBM in a dose escalating phase I/IIa trial. In arm 1 including groups 1 and 3, ParvOryx was intratumorally injected as the first dose for the treatment. In arm 2 having group 2, patients were given five intravenous doses of the virus from 1 to 5 days of infusion. Patients from all the groups underwent tumor resection on day 10 followed by virus infusion around the resection cavity. Pharmacokinetics analysis showed the presence of viral genomes (Vg) and infectious particles in blood at measurable concentrations. Continuous increase in the levels of blood Vg was observed during each post intravenous infusion. After 22hr post-infusion, Vg levels decreased by two orders of magnitude. ParvOryx transcripts were detected in the resected tumors in 4 out of 6 patients receiving intravenous ParvOryx, suggesting the ability of the virus to cross the blood brain/tumor barrier. A median OS of 15.5 months was observed irrespective of the route of delivery. Additionally, CD8+ and CD4+ T cells successfully infiltrated tumors as studied in biopsy samples from 6 patients (57).

### Toca 511

Vocimagene amiretrorepvec, a murine leukemia virus expresses a yeast gene encoding cytosine deaminase converting an antifungal drug, 5-fluorocytosine (5-FC) into 5-fluorouracil, an antimetabolite drug. Patients with HGG who recurred after receiving standard therapy had surgical resection in this ascending-dose phase I study of Toca 511 (NCT01470794). Toca 511 was injected into the wall of the resection cavity, and cycles of Toca FC were then taken orally. Durable responses were seen in 21% of patients and durable responders showed robust survival of 33.9 to 52.2 months after Toca 511 infusion (58). For a multicentric phase II/III trials conducted across 58 centers, 403 patients were enrolled to study the efficacy, and 400 patients were enrolled for safety studies. Toca 511 was injected into the resection cavity wall of the patients intraoperatively, followed by Toca FC oral dose for six weeks after surgery. The primary endpoint of improved OS of the trial could not be achieved. The OS was 11.1 months. However, the safety studies demonstrated its safety profile. It did not provide additional survival benefits over standard care of treatment (59).

### Measles virus human carcinoembryonic antigen

Edmonston strain of Measles is engineered to express reporter transgene encoding the human carcinoembryonic antigen. In a phase I study in 23 patients, MV CEA administered *via* intratumoral route *a priori* before *en block* tumor resection or in the resection cavity showed a median OS of 11.4 and 11.8 months, respectively (60).

## Impediments in the clinical implementation of oncolytic virus therapy in high-grade gliomas

Oncolytic virus therapy for gliomas is evolving, both preclinical and phase II clinical studies show significant efficacy and favorable safety profiles. Despite several clinical trials exploring the use of OV’s in gliomas, there are still certain fundamental challenges in translating the promising results in early clinical trials. These challenges include;

1. Modulating OVs-mediated host immune response and long-lasting anti-tumor response.
2. Absence of discerning features of radiographic response to OV therapy-induced pseudo progression from actual disease progression.
3. Finding appropriate markers for therapeutic efficacy.
4. Overcoming existing obstacles in OVs delivery with minimally invasive administration.
5. Physical barriers in and around the tumor microenvironment impede ineffective replication of the virus in the tumor resulting inefficient tumor cell kill.

## Therapeutic potential of oncolytic virotherapy for high-grade gliomas: The future directions

Oncolytic Virotherapy is associated with a unique regulatory safety profile and challenges including tumor-targeted specificity, disruption of the immunosuppressive tumor environment, and robustness of the immune system. Various clinical trials have shown the effectiveness of OV therapy with immunotherapeutic molecules, this has been made possible by the change in the phenotype of T cells from exhausted to activated type (61).

As discussed in the previous section “Modulating OVs-Mediated Host Immune Responses”, is a tricky issue wherein, OVs can not only trigger the immune system but also activate anti-virus immune responses. Moreover, different viruses behave differently within the tumor milieu. This might be the main reason that the efficacy of OVTs as monotherapies, is not very satisfactory. Therefore, it is important to find more synergistic combination therapies and understand the underlying mechanisms of each therapy on immune response and subsequently their clinical effects (62) (63).

As discussed, one of the challenges in OVT is to deliver the OVs at the targeted site, one of the methods to accurately deliver the OVs is by Convention enhanced delivery (CED) (64). CED of OV’s allows effective distribution of bigger volumes over large tumor areas (65). CED is being tested in phase I and II clinical trials to enhance the transduction efficiency of viral vectors. Another approach for effectual delivery of OVs is stem cell-based (neural and mesenchymal stem cells) delivery of oncolytic virotherapy. This approach shields the therapeutic viruses from the innate immune system and, at the same time, efficiently increases viral distribution to distant tumor areas.

For OVT to be specific & successful, careful screening of patients based on genetic mutations and protein expressions in their tumors should be implemented. By implementing genetic engineering approach, targeting, and detargeting can be achieved by modification of the outer membranes in enveloped viruses and the capsid of the naked viruses. Additionally, adaptor molecules are used to mask the cell binding domain of the capsid, genetic modification of capsid protein targeting cellular receptors, and insertion of the ligands (66–68). Moreover, viruses are armed with cytotoxic tools to enhance the induced immune response. Various therapeutic genes are considered to improve the cytotoxicity of OVs. To enhance the efficacy of chemotherapy, the target cell is sensitized with OV expressing enzyme, which activates the prodrugs. Similarly, tumor radiation can be induced by ion transport proteins, and immunostimulatory factors can generate immune responses (69).

Well-designed clinical trials, including a synergistic combination of OVT and immunotherapy, will pave the way for effective and specific OV glioma treatments.

## Conclusion

Viruses have manifested to acquire multifaceted tumor-killing mechanisms. They are now emerging as promising agents to modulate the immune response within the tumor by implementing the strategies to use viruses with the capability to cross the blood-brain barrier and/or by direct tumor injection to enhance the safety, tumor-specific replication and to boost the immune system by using various gene editing strategies. Modifications are also required not only to reduce the adverse effects, and treatment-associated toxicity but also to improve the remission rates in glioma patients.

Various preclinical and clinical studies have been designed using oncolytic virus therapies as a monotherapy or as a combinational approach, including immune checkpoint inhibitors or adoptive cell therapy, or cytokines. These combinatorial strategies are being explored in various clinical studies to improve the survival outcomes in high-grade gliomas. The potential application of OV’s either as monotherapy or combinatorial therapy should be explored rigorously in clinical trials not only for their clinical efficacy but also for their safety profile in high-grade gliomas to translate this novel treatment platform into routine clinical practice.

## Author contributions

SA and AC - conceptualized, wrote the article and review of the article. JG – made substantial contributions to conceptualization, writing and edited the article. SY and GC made contributions to tables and figures. RP supervised, conceptualized and edited the article. All authors contributed to the article and approved the submitted version.
